# Correction: Oxidative carboxylation of olefins with CO_2_: environmentally benign access to five-membered cyclic carbonates

**DOI:** 10.1039/d0ra90023k

**Published:** 2020-03-16

**Authors:** Liang Wang, Sisi Que, Ziwei Ding, Esmail Vessally

**Affiliations:** State Key Lab of Coal Mine Disaster Dynamics and Control, Chongqing University Chongqing 400044 China; State Key Laboratory of Coal Resources in Western China, Xi’an University of Science and Technology Xi’an 710054 China; Key Laboratory of Hydraulic and Waterway Engineering of the Ministry of Education, College of River and Ocean Engineering, Chongqing Jiaotong University Chongqing 400074 China; Department of Chemistry, Payame Noor University Tehran Iran vessally@yahoo.com

## Abstract

Correction for ‘Oxidative carboxylation of olefins with CO_2_: environmentally benign access to five-membered cyclic carbonates’ by Liang Wang *et al.*, *RSC Adv.*, 2020, **10**, 9103–9115.

The Royal Society of Chemistry regrets that incorrect details were given for Ref. 10b, 11b and 11c in the original article. The correct versions of Ref. 10b, 11b and 11c are given below as Ref. 1, 2a and 2b, respectively.

The Royal Society of Chemistry apologises for these errors and any consequent inconvenience to authors and readers.

## Supplementary Material
